# Efficacy of liraglutide, a glucagon-like peptide-1 (GLP-1) analogue, on body weight, eating behavior, and glycemic control, in Japanese obese type 2 diabetes

**DOI:** 10.1186/1475-2840-11-107

**Published:** 2012-09-14

**Authors:** Yuya Fujishima, Norikazu Maeda, Kana Inoue, Susumu Kashine, Hitoshi Nishizawa, Ayumu Hirata, Junji Kozawa, Tetsuyuki Yasuda, Kohei Okita, Akihisa Imagawa, Tohru Funahashi, Iichiro Shimomura

**Affiliations:** 1 Department of Metabolic Medicine, Graduate School of Medicine, Osaka University, 2-2-B5, Yamada-oka, Suita, Osaka, 565-0871, Japan

**Keywords:** Liraglutide, Glucagon-like peptide-1 (GLP-1), Obesity, Eating behavior, Diabetes, Incretin

## Abstract

**Background:**

We recently reported that short-term treatment with liraglutide (20.0 ± 6.4 days) reduced body weight and improved some scales of eating behavior in Japanese type 2 diabetes inpatients. However, it remained uncertain whether such liraglutide-induced improvement is maintained after discharge from the hospital. The aim of the present study was to determine the long-term effects of liraglutide on body weight, glycemic control, and eating behavior in Japanese obese type 2 diabetics.

**Methods:**

Patients with obesity (body mass index (BMI) >25 kg/m^2^) and type 2 diabetes were hospitalized at Osaka University Hospital between November 2010 and December 2011. BMI and glycated hemoglobin (HbA1c) were examined on admission, at discharge and at 1, 3, and 6 months after discharge. For the liraglutide group (BMI; 31.3 ± 5.3 kg/m^2^, n = 29), patients were introduced to liraglutide after correction of hyperglycemic by insulin or oral glucose-lowering drugs and maintained on liraglutide after discharge. Eating behavior was assessed in patients treated with liraglutide using The Guideline For Obesity questionnaire issued by the Japan Society for the Study of Obesity, at admission, discharge, 3 and 6 months after discharge. For the insulin group (BMI; 29.1 ± 3.0 kg/m^2^, n = 28), each patient was treated with insulin during hospitalization and glycemic control maintained by insulin after discharge.

**Results:**

Liraglutide induced significant and persistent weight loss from admission up to 6 months after discharge, while no change in body weight after discharge was noted in the insulin group. Liraglutide produced significant improvements in all major scores of eating behavior questionnaire items and such effect was maintained at 6 months after discharge. Weight loss correlated significantly with the decrease in scores for recognition of weight and constitution, sense of hunger, and eating style.

**Conclusion:**

Liraglutide produced meaningful long-term weight loss and significantly improved eating behavior in obese Japanese patients with type 2 diabetes.

## Introduction

The worldwide epidemic of type 2 diabetes has been fueled by the increase in the number of obese and overweight people. Similar to Western countries, in East Asian countries, the risk of death from cancer and cardiovascular diseases has increased in parallel with the increase in body mass index (BMI) [1]. The World Health Organization (WHO) estimates that 2.3 billion adults will be overweight and >700 million will be obese by 2015 [2]. Effective therapeutic strategy for obese type 2 diabetes should be developed without delay, but it is often difficult to control appetite and to maintain body weight in obese type 2 diabetes patients. Intensive insulin therapy may result in fine glycemic control and prevent microvascular complications, but such treatment usually increases body weight [3]. In addition, the oral glucose-lowering agents sulfonylurea (SU) and thiazolidinedione also increase body weight by enhancing glucose uptake into adipocytes.

Liraglutide, a glucagon-like peptide (GLP-1) analogue, is a member of new classes of anti-diabetic agents and is characterized by induction of insulin secretion only during hyperglycemia as an incretin effect. The Liraglutide Effect and Action in Diabetes (LEAD) studies have demonstrated a significant weight reduction by liraglutide [4], but such liraglutide-mediated weight reduction has not been observed in Japanese type 2 diabetes patients [5-7]. Subjects of the LEAD trial were obese type 2 diabetic patients, while the Japanese subjects enrolled in the liraglutide trial were non-obese type 2 diabetes patients (BMI; 23.5-25 kg/m^2^). In a pilot study, we recently reported that short-term liraglutide treatment reduced BMI, waist circumference, and visceral fat area, and reduced the scale for eating behavior in Japanese type 2 diabetes inpatients (age; 61.2 ± 14.0 years, BMI; 28.3 ± 5.2 kg/m^2^, duration of diabetes; 16.9 ± 6.6 years) [8]. However, this short-term study was performed only during hospitalization and thus it remains uncertain whether these effects of liraglutide are maintained after discharge. In the present study, we investigated the effect of liraglutide on body weight, glycemic control, and eating behavior until 6 months after discharge in Japanese obese patients (BMI >25 kg/m^2^) with type 2 diabetes.

## Materials and methods

### Subjects and clinical examination

The study subjects were selected among patients hospitalized at the Division of Endocrinology and Metabolism of Osaka University Hospital between November 2010 and December 2011. All patients were hospitalized to undergo medical treatment for diabetes. The inclusion criteria were as follows: (1) type 2 diabetes with obesity (BMI >25 kg/m^2^) at admission; (2) patients continued to visit Osaka University Hospital for treatment after discharge; (3) patients were treated with liraglutide or insulin at discharge and continued to be treated with the same regimen after discharge. Physical examination and various metabolic parameters were measured on admission. Body weight and HbA1c were examined at 1, 3, and 6 months after discharge. The selection of the glucose-lowering agent for treatment was left to the attending physician. In the liraglutide group, each patient was treated with insulin or oral glucose-lowering drugs under diet therapy after admission. After achieving the target levels of glycemic control [fasting plasma glucose (FPG) <150 mg/dL and postprandial 2-h plasma glucose <200 mg/dL], treatment with insulin or oral glucose-lowering agents was replaced with liraglutide at 0.3 mg/day, which was increased by 0.3 mg/day every one week to a final dose of 0.9 mg/day, representing the maximum dose used in Japan. The introduction of liraglutide and increase in liraglutide dosage was decided by the attending physician. In the insulin group, each patient was treated with insulin during hospitalization and glycemic control was maintained by insulin treatment after discharge.

Written consent was obtained from each subject after explaining the purpose and possible complications of the study. The study protocol was approved by the human ethics committee of Osaka University and was registered with the University hospital Medical Information Network (Number: UMIN 000004192).

### Questionnaire for eating behavior

Eating behavior was assessed in patients on liraglutide treatment by using the questionnaire of The Guideline For Obesity issued by the Japan Society for the Study of Obesity, at admission, discharge, 3 and 6 months after discharge. As reported previously [8], this questionnaire consists of 55-item questions of seven major scales as follows: 1) Recognition for weight and constitution (e.g., ‘Do you think it is easier for you to gain weight than others?’), 2) External eating behavior (e.g., ‘If food smells and looks good, do you eat more than usual?’), 3) Emotional eating behavior (e.g., ‘Do you have the desire to eat when you are irritated?’), 4) Sense of hunger (e.g. ‘Do you get irritated when you feel hungry?’), 5) Eating style (e.g., ‘Do you eat fast?’), 6) Food preference (e.g., ‘Do you like meat?’), 7) Regularity of eating habits (e.g., ‘Is your dinner time too late at night?’). All items were rated on a four-point scale ranging from 1 (seldom) to 4 (very often).

### Statistical analysis

The Student's t-test and χ^2^ test were used to compare baseline characteristics of the liraglutide and insulin groups. Variables with skewed distribution were analyzed by the Mann–Whitney *U*-test. The Student's t-test was used for comparison of results obtained at admission, discharge, and post-discharge. In all cases, P values <0.05 were considered statistically significant. All analyses were performed using the JMP software (JMP 8.0; SAS Institute Inc., Cary, NC).

## Results

### Characteristics of participants

Table 1 shows the baseline characteristics of 29 subjects on liraglutide and 28 subjects on insulin treatment. For the liraglutide group, the mean BMI was 31.3 kg/m^2^, glycated hemoglobin (HbA1c) was 8.5%, and fasting C-peptide was 2.09 ng/mL. Among the patients of the liraglutide group, 55% were treated with insulin at admission. The baseline characteristics of patients of the insulin group were similar to those of the liraglutide group, but HbA1c levels and frequencies of insulin treatment before admission were higher in the insulin group compared to the liraglutide group. The duration of diabetes and fasting C-peptide level were similar in both groups. Thus, the enrolled patients were obese Japanese subjects, had relatively long duration of diabetes, and most of them were treated with insulin, but had preserved insulin secretion capacity.

**Table 1:** Baseline characteristics

	**Liraglutide (n=29)**	**Insulin (n=28)**	***P*value**
Males/females	12/17	14/14	0.514
Age (years)	58.1 ± 12.1	63.9 ± 12.2	0.080
Duration of diabetes (years)	16.0 ± 8.3	18.5 ± 10.0	0.309
Body weight (kg)	82.0 ± 16.2	76.6 ± 15.5	0.122
Body mass index (kg/m^2^)	31.3 ± 5.3	29.1 ± 3.0	0.102
HbA1c (%)	8.5 ± 1.4	9.4 ± 1.6	0.023
Fasting plasma glucose (mg/dL)	159.0 ± 49.7	156.4 ± 59.2	0.655
Fasting C-peptide (mg/dL)	2.09 ± 0.97	1.97 ± 0.24	0.267
Low-density lipoprotein-cholesterol (mg/dL)	113.1 ± 36.5	116.6 ± 28.2	0.689
High-density lipoprotein-cholesterol (mg/dL)	44.2 ± 8.1	46.1 ± 13.8	0.534
Triglycerides (mg/dL)	149.4 ± 55.2	201.2 ± 145.0	0.432
Hypertension (%)	83	86	0.760
Dyslipidemia (%)	90	86	0.650
Previous treatment			
Biguanide (%)	38	18	0.092
Sulfonylurea (%)	31	21	0.410
Alpha-glucosidase inhibitor (%)	17	29	0.308
Thiazolidinedione (%)	21	14	0.525
DPP-IV inhibitor (%)	10	7	0.669
Glinide (%)	3	0	0.322
Insulin (%)	55	82	0.029

Data are mean ± SD or number of subjects.

The number of patients treated at discharge with liraglutide at 0.3, 0.6, and 0.9 mg/day was 3, 11, and 15 patients, respectively. The total daily dose (TDD) of insulin was 0.59 unit/kg/day at discharge in the insulin group. In the liraglutide group, the duration of hospitalization was 30.8 ± 9.3 days and the time from liraglutide induction to discharge was 20.0 ± 6.9 days. In the insulin group, the duration of hospitalization was 29.1 ± 8.4 days, indicating similar duration of hospitalization in the two groups. At 6 month after discharge, the number of patients treated with liraglutide at 0.3, 0.6, and 0.9 mg/day was 3, 7, and 19 patients, and SU and biguanide was used in 10 and 15 patients of the liraglutide group, respectively. In the insulin group, SU, biguanide, and alpha-glucosidase inhibitor was used by 1, 9, and 3 patients at 6 month after discharge, respectively. There were no differences in the frequency of hypoglycemic episode between the liraglutide and insulin groups.

### Changes in body weight and HbA1c

**Figure 1:**
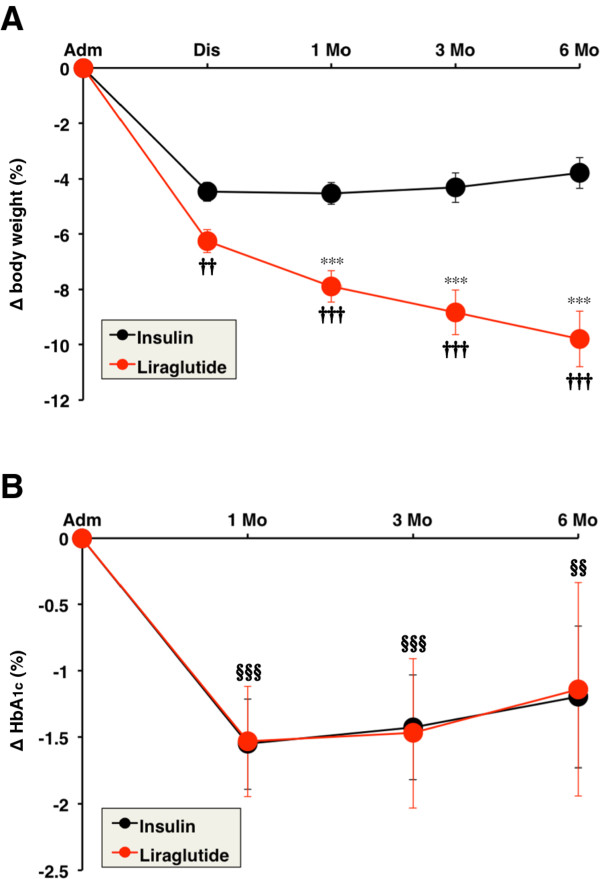
**Serial changes in body weight (A) and HbA1c (B) induced by insulin and liraglutide.** Data are mean ± SEM. Adm, admission; Dis, discharge; Mo, month. ***P < 0.001, compared with the values at discharge. †† *P* < 0.01, ††† *P* < 0.001, compared with the values of the insulin group. §§ *P* < 0.01, §§§ *P* < 0.001, compared with the values at admission.

Body weight and HbA1c were measured during hospitalization and after discharge. In the liraglutide group, BMI at admission, discharge, 1, 3, and 6 months after discharge were 31.3 ± 5.3, 29.3 ± 4.8, 28.8 ± 4.6, 28.5 ± 4.4, and 28.2 ± 4.3 kg/m^2^, respectively. Liraglutide treatment significantly decreased BMI even after 6 months from discharge compared to admission (*P* < 0.001). Figure 1A shows changes in body weight from admission in both groups. In the insulin group, body weight was significantly reduced from admission to discharge while there were no changes in body weight from discharge to 6 months (Figure 1A). Changes in body weight at 1, 3, and 6 months after discharge were significantly larger in the liraglutide group than in the insulin group after adjustment for age, sex, BMI, HbA1c, and insulin treatment at admission (*P* < 0.001). Among the subjects treated with liraglutide, the change in body weight at 6 months after discharge correlated significantly with BMI at admission (*P* = 0.002).

HbA1c was significantly reduced at 1 month from discharge but tended to increase at 6 months after discharge in both groups (Figure 1B). Liraglutide treatment significantly reduced the mean HbA1c by −1.1% at 6 months after discharge compared to admission. Insulin administration also significantly decreased HbA1c, and the reduction in HbA1c was similar in both liraglutide and insulin group. After adjustment for age, sex, BMI, HbA1c, and insulin treatment at admission, the decrease in HbA1c was significant only at 1 month after discharge in the liraglutide group compared with the insulin group (*P* < 0.05).

### Assessment of eating behavior

**Figure 2:**
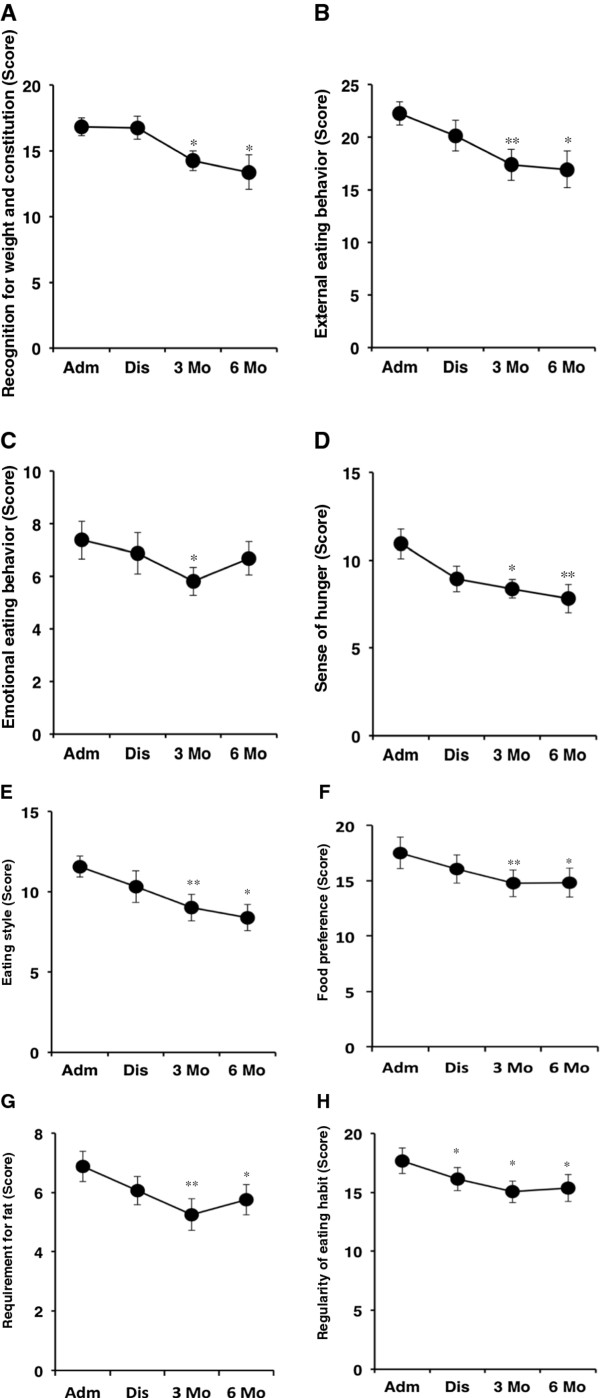
**Changes in the scores of eating behavior induced by liraglutide.** Eating behavior was assessed in 16 patients (8 males and 8 females) using a questionnaire as described in the Methods section. Data are mean ± SEM. *P < 0.05, **P < 0.01, compared with the values at admission.

Next, the effect of liraglutide on eating behavior was evaluated in 16 subjects (8 males and 8 females) until 6 months after discharge. Figure 2 depicts the scores of the questionnaire for eating behavior at admission, discharge, 3 months, and 6 months after discharge. The scores for recognition of weight and constitution (Figure 2A) and external eating behavior (Figure 2B) were not changed at discharge, but were significantly decreased at 3 and 6 months after discharge. Emotional eating behavior was reduced at 3 month after discharge but tended to deteriorate at 6 month after discharge (Figure 2C). Importantly, liraglutide treatment promptly reduced the score for sense of hunger and markedly suppressed the sense of hunger (Figure 2D). The score for eating style decreased gradually and was significantly reduced at 3 and 6 months after discharge (Figure 2E). Interestingly, the score for food preference (Figure 2F), especially requirement for fat (Figure 2G), was significantly decreased by liraglutide at 3 and 6 months after discharge. Liraglutide treatment reasonably reduced the score for regularity of eating habit at discharge and maintained the same reduction until 6 months after discharge (Figure 2H).

Next, we examined the correlations between changes in body weight and eating behavior from admission to 6 months after discharge. The decrease in total score of eating behavior correlated significantly with the extent of weight loss (*P* = 0.029, R = 0.48). Interestingly, weight loss correlated significantly with the decrease in the scores for recognition of weight and constitution (*P* = 0.007, R = 0.57), sense of hunger (*P* = 0.021, R = 0.50), and eating style (*P* = 0.037, R = 0.46). The decrease in the score for external eating behavior tended to be associated with weight reduction (*P* = 0.061, R = 0.42). The scales for other parameters of eating behavior did not correlate significantly with weight reduction.

**Figure 3:**
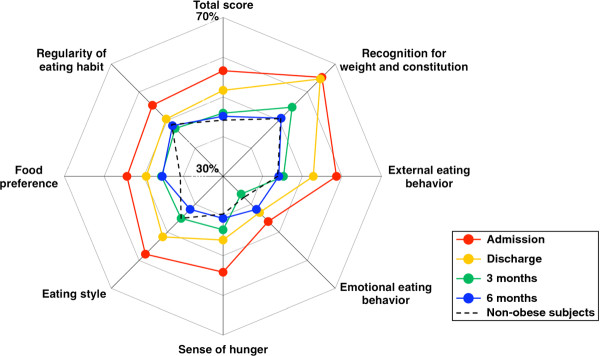
**Liraglutide-induced improvement in eating behavior.** Changes in the scores of eating behavior were expressed as a radar chart.

Figure 3 shows a radar chart of the eating behavior. The black dotted line represents the scores of non-obese healthy subjects. All scales for eating behavior were disordered at admission (red line), but these parameters tended to show improvement at discharge (yellow line). Furthermore, all scales showed further improvement and the scores of eating behavior moved towards those of the non-obese healthy subjects at 3 (green line) and 6 (blue line) months after discharge.

## Discussion

The major findings of the present study were: 1) liraglutide treatment effectively reduced body weight up to 6 months after discharge compared to conventional insulin therapy in Japanese obese patients with type 2 diabetes; 2) liraglutide-induced reduction in HbA1c was similar to that observed with insulin treatment; 3) maintained improvement in eating behavior up to 6 months after discharge in patients on liraglutide treatment with various scales gradually moving to those recorded in non-obese normal subjects.

The LEAD-5 study demonstrated that liraglutide decreased HbA1c similar to ultra-long acting insulin analogue, glargine, and also significantly reduced body weight compared with glargine [9]. In the present retrospective study, we compared the effects of liraglutide and insulin on body weight and HbA1c. Almost all patients of the insulin group received intensive insulin therapy at discharge, and the TDD of insulin was relatively high (0.59 unit/kg/day). Significant body weight reduction was observed in both liraglutide and insulin groups during hospitalization and this was due to the in-hospital diet therapy. However, weight reduction was persistently observed after discharge only in the liraglutide group. Liraglutide treatment may be distinguished from the intensive insulin therapy by effective reductions in body weight and HbA1c. Further studies are needed to determine the differences in the therapeutic effects of liraglutide and intensive insulin therapy. The combination treatment of GLP-1 receptor agonist and basal insulin has been also approved in the USA and EU [10], but is yet to be approved in Japan. The effect of such combination therapy on body weight needs to be examined in the future.

GLP-1 promotes satiety and reduces food intake [11,12], but the effect of GLP-1 treatment on eating behavior has not been fully examined in human subjects with type 2 diabetes. We reported recently that short-term treatment with liraglutide did not only reduce food intake and visceral fat adiposity but also decreased the scores of certain parameters of eating behavior during hospitalization [8]. However, we did not provide evidence for the long-term effects of liraglutide on eating behavior. Interestingly, the scores of obese type 2 diabetic patients at admission were higher in all major scales than non-obese subjects (Figure 3). Liraglutide administration decreased these scores and maintained such improvement in eating behavior, with the exception of the scale of emotional eating behavior, until 6 months after discharge, and the total score of eating behavior of some liraglutide-treated patients moved closer to that of non-obese healthy subjects. GLP-1 delays gastric emptying and induces satiety, which is probably related to the combined effect of GLP-1 on the gastrointestinal tract and the brain, leading to decreased energy intake and weight reduction [13,14]. Furthermore, the improvement in the scale for eating style by liraglutide may be partly accounted for by GLP-1-induced suppression of gastrointestinal peristalsis. The extent of weight reduction at 6 months after discharge correlated significantly with the reduction in the scores for sense of hunger and eating style, suggesting that the liraglutide-induced weight reduction is mediated partly through improvement in eating behavior.

A recent meta-analysis of weight reduction by GLP-1 receptor agonists showed that administration of these agents resulted in 3% weight reduction at 6 months [15]. The LEAD-5 trial showed 2.1% weight reduction under treatment with liraglutide at 1.8 mg/day [9]. Further, another study, which used liraglutide at up to 3.0 mg/day for 20 weeks for treatment of obesity (BMI; 30–40 kg/m^2^), reported 7.4% weight loss [16]. In present study, liraglutide administered at the maximum dose of 0.9 mg/day achieved about 10% body weight reduction at 6 months after discharge. This result far exceeds those of previous studies [9,15,16]. What are the reasons for the excellent results noted in the present study? 1) Patients treated with liraglutide were also placed on strict diet therapy and received a special educational program for weight reduction during hospitalization over 30.8 ± 9.3 days. The importance of these two factors is evident from the rate of weight reduction in the liraglutide group achieved in the present study; weight reduction of −6.5% during hospitalization and only −3.5% between discharge and 6 months after discharge. 2) It is possible that GLP-1 analogue exhibited its utmost effectiveness on weight reduction, because glucose toxicity was corrected mainly by insulin therapy before induction of liraglutide. A poor response to GLP-1 has been described in patients with poorly controlled diabetes but this is reversed following normalization of glycemic control [17,18]. High levels of serum GLP-1 were also reported in patients with the metabolic syndrome [19], suggesting the existence of “GLP-1 resistance”. Our group has also demonstrated low expression levels of GLP-1 receptor mRNA in the islets of obese mice, which were significantly reversed after glucose-lowering medications [20].

GLP-1 receptor agonists do not only have glucose-lowering effect but also improve other metabolic parameters such as lipid profile and blood pressure following 5-10% weight reduction [21]. Long-term treatment with exenatide, a GLP-1 receptor agonist, significantly improved cardiovascular risk factors, such as LDL-C, HDL-C, and triglyceride, and biomarkers of liver function [22]. Furthermore, treatment with GLP-1 improved postprandial hyperlipidemia, suggesting that GLP-1 could reduce cardiovascular disease risk in type 2 diabetes [23]. In present study, liraglutide significantly decreased LDL-C at 6 months after discharge (admission: 113.1 ± 36.5, at 6 months after discharge: 95.3 ± 22.9 mg/dL, *P* =0.03) although 66% of the patients were treated with statins at admission. As shown in Figure 2G, the score for requirement for fat decreased after liraglutide, suggesting that reduction of dietary fat intake partly contributed to the observed decrease in LDL-C. However, the questionnaire did not contain questions about the types of fat consumed, e.g. saturated fat or non-saturated fat, and daily frequency or quantity of fat intake. Questions for food preference would be further improved in the future. Several studies have described the extrapancreatic actions of GLP-1, especially its beneficial effects on the cardiovascular system [2]. Treatment with GLP-1 improved endothelial dysfunction in type 2 diabetics with ischemic heart diseases [24]. We recently showed that short-term liraglutide treatment decreased serum high-sensitivity C-reactive protein (hsCRP) and soluble intracellular adhesion molecule-1 (sICAM-1) levels [8]. Long-term prospective studies are needed to determine whether the observed changes in obesity, eating behavior, and various metabolic parameters induced by treatment with liraglutide translate into protection against cardiovascular events in obese type 2 diabetics.

The present study has several limitations. The study was not a randomized clinical trial (RCT) and not prospective in design. The baseline characteristics of the patients of the liraglutide and insulin groups were not identical. In addition, the present study included a relatively small population. Furthermore, assessment of eating behavior could not been performed in the insulin group.

In summary, treatment with liraglutide effectively reduced body weight and improved eating behavior in obese Japanese patients with type 2 diabetes for up to 6 months. Liraglutide is potentially useful for the treatment of obese patients with type 2 diabetes. The weight-lowering effects of liraglutide should be examined in more detail in the future in a double-blind placebo-controlled clinical trial.

## Competing interests

The authors declare no competing interests.

## Authors’ contributions

YF acquired and analyzed data, and wrote the manuscript. NM conceived study, analyzed data, and wrote the manuscript. KI, SK, HN, AM, JK, TY, KO, and AI acquired and researched data. TF and IS reviewed the manuscript. All authors read and approved the final manuscript.
